# Efficacy of alternative cooling devices used for insulin storage without refrigeration under hot-humid environment

**DOI:** 10.1080/07853890.2022.2067355

**Published:** 2022-04-21

**Authors:** Supang Taerahkun, Chutintorn Sriphrapradang

**Affiliations:** Department of Medicine, Faculty of Medicine Ramathibodi Hospital, Mahidol University, Bangkok, Thailand

**Keywords:** Diabetes mellitus, drug storage, hot temperature, insulin therapy, temperature

## Abstract

**Background:**

Insulin is temperature sensitive as high temperatures reduce its potency. Refrigeration for insulin storage is still needed but households in remote areas do not have refrigerators. Also, the electricity supply is usually affected by natural disasters. We aim to examine the temperature-reducing efficacy of cooling devices in hot-humid conditions.

**Methods:**

Five cooling devices, (1) earthen jar filled with water, (2) earthen jar filled with soil, (3) two clay pots, gap filled with wet soil, (4) two clay pots, gap filled with wet sand, and (5) commercial cooling wallet were used in this study. External and internal temperatures were monitored by the temperature logger between October 2019 and September 2020 in Narathiwat, Thailand. Cooling efficacy was assessed by average absolute temperature reduction and relative cooling effect.

**Results:**

Mean external temperature and humidity were 27.3 ± 1.5 °C and 78.2 ± 7.1%RH. The mean differences between the external and internal temperatures were; device (1) −0.1 ± 0.6 °C (*p* = NS), (2) 0.0 ± 0.8 °C (*p* = NS), (3) −1.7 ± 0.9 °C (*p* < .0001), (4) −2.0 ± 0.9 °C (*p* < .0001), and (5) −1.8 ± 0.9 °C (*p* < .0001). Device no. 3, 4, and 5 achieved a constant temperature reduction. The most efficacious device was device no. 4 with a relative cooling effect of 63.6% better than the cooling wallet (57.7%, *p* = .003). All devices were more efficacious at lower humidity levels.

**Conclusions:**

Traditional low-cost devices, such as clay pots, reduce storage temperatures to or close to room temperature in hot-humid climates. This study provides some guidance for insulin storage in hot-humid environments.

## Introduction

Insulin therapy is the mainstay of treatment for those who have diabetes mellitus type 1 or type 2 with advanced disease. Due to the biological activity of insulin, loss of insulin effectiveness could be from exposure to a temperature above 32 °C [1–3]. This can lead to deterioration in glycemic control [4,5]. In-use insulin can be stored at room temperature not to exceed 25 or 30 °C, within a range from 10 days to 8 weeks. It should never be frozen or kept in direct sunlight [1,2]. The East Africa Diabetes Study Group (EADSG) recommended keeping the insulin in use at room temperature (20–25 °C) for 6 weeks, avoiding sunlight, and for 4 weeks if the temperature goes up to 30 °C [1,6].

In Thailand, the mean temperature throughout 2020 was 28 °C with the highest temperature of 43.5 °C [7]. Refrigeration for insulin storage is needed in countries with hot climates. Unfortunately, in many countries, a large proportion of patients with diabetes mellitus who live in rural areas lack refrigeration facilities in their homes [8–11]. While insulin storage requires a constant electricity supply, this cannot be assured, especially during natural disasters, such as earthquakes, floods, typhoons, and hurricanes [12].

Without mechanical refrigeration, evaporative cooling is a well-known and efficient system for maintaining temperature for short-term storage of perishables [13]. The traditional evaporative cooler consists of a porous outer clay pot lined with water or wet sand containing an inner pot within which the food is placed. The device cools as the water evaporates and draws the heat from the inner pot. These coolers were proved for short-term insulin storage [14–16]. The evaporative cooling system does not require electricity and can be easily constructed using locally available materials with unskilled labour. However, high ambient humidity condition decreases the cooling capacity of the evaporative cooler since the water cannot evaporate well under these conditions. To date, there have been few studies on the cooling efficacy of insulin storage devices, which were performed in desert climates countries [15,16]. Noteworthy, there is no data in tropical regions, such as Southeast Asia countries. Therefore, we sought to develop insulin storage devices that could be easily reproducible using locally available materials at minimal cost in Thailand.

## Materials and methods

We hypothesized that the traditional low-cost devices with an effective evaporative cooling system have a comparable reducing temperature capacity with a commercial cooling device. To easily compare the results, we conducted an experimental study similar to a previous landmark study by Ogle and colleagues [16].

### Cooling devices

**Figure 1.**
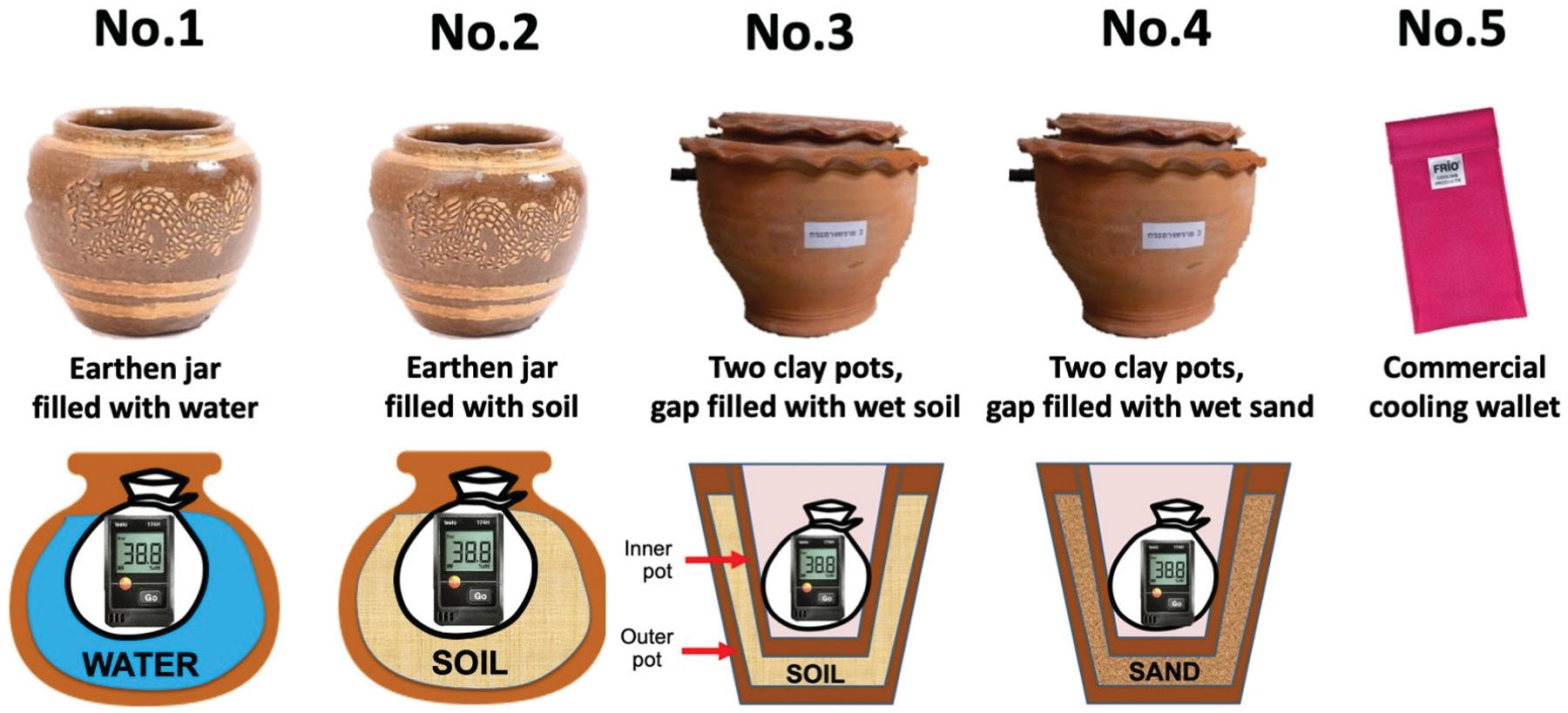
Illustration of cooling devices.

There are five varieties of devices used in this study: (1) earthen jar (porous and glazed) filled with water, (2) earthen jar (porous and glazed) filled with soil, (3) two clay pots (porous and unglazed), gap filled with wet soil, (4) two clay pots (porous and unglazed), gap filled with wet sand, and (5) commercial cooling wallet (FRIO^®^, Haverfordwest, UK). All varieties of devices were named as devices no. 1 to no. 5, respectively. Figure 1 and Table 1 describe the characteristics of each device.

**Table 1.** Description of cooling devices.

No.	Cooling devices	Dimensions	Descriptions	Comments
1	Earthen jar filled with water	26 × 35 cm	Porous, glazed jar filled with water, covered by an aluminium lid	Level of water about 10 cm below the edge of the jar
2	Earthen jar filled with soil	26 × 35 cm	Porous, glazed jar filled with wet soil, covered by an aluminium lid	Level of soil about 10 cm below the edge of the jar
3	Two clay pots, gap filled with wet soil	Inner: 25 × 26 cm Outer: 33.5 × 28 cm	Porous, unglazed clay pot, placing a smaller clay pot into a larger clay pot.	Wet soil is placed between the interior of the large pot and the exterior surface of the small pot, covered by damp cloth
4	Two clay pots, gap filled with wet sand	Inner: 25 × 26 cm Outer: 33.5 × 28 cm	Porous, unglazed clay pot, placing a smaller clay pot into a larger clay pot	Wet sand is placed between the interior of the large pot and the exterior surface of the small pot, covered by damp cloth
5	Commercial cooling wallet (FRIO^®^)	14 × 15 cm	FRIO^®^ cooling wallet, suspended	Wet before each study period

### Study design and setting

The study was conducted from October 2019 to September 2020 at Narathiwat (first author’s hometown, Latitude: 6°25′35″N, Longitude: 101°49′23.09″E), Thailand. Narathiwat, the extreme southern city of Thailand, has a tropical monsoon climate. Since Narathiwat is near the equator, the temperature variation is slight throughout the year. Despite abundant rainfall, the dry season is short, starting from February until April. Heavy rainfall is from November to December. The experiment was conducted in an internally well-ventilated internal room, out of direct sunlight. The cooling devices were placed at least 80 cm apart in a grid arrangement and spaced out from walls or other objects to maximize airflow. Before the beginning of the testing period, the devices were left at a constant temperature for 2 h to ensure thermal stabilization. Water refilling was required every 24-h apart to maintain the evaporation effect of all devices.

### Temperature and humidity measurements

The temperature and humidity of each device and external air were monitored using a TestoR174H high-accuracy temperature and humidity data logger (Westchester, PA, USA) [17]. These loggers were programmed to start recording at a preset time. Before placement in the cooling devices, the loggers were placed together in the same environment about 1 h before the recording. The logger clocks were synchronized to achieve simultaneous monitoring of temperature. All observations were made every 5 min intervals for 24 h, then the same experiment was repeated in the next 24 h for a total experimental time of 72 h every month for 12 months. The cooling efficacy was evaluated by average absolute temperature difference and relative cooling effect.

Instead of insulin, the thermometer filled in the plastic bag was placed in each device. The actual vials of insulin were not necessary for the experiment. To ensure the reliability of the thermometers, the mean deviance of each thermometer was recorded and calculated for inter-thermometer variability.

In addition, the cooling efficacy according to the capacity for evaporative heat loss was assessed by saturated water vapour pressure and absolute humidity inside the device. Since higher saturated water vapour pressure and absolute humidity slow evaporation, saturated water vapour pressure and absolute humidity were calculated by the equations below.

#### Saturated water vapour pressure (e_sat_) by Goff-Gratch equation


$$\log_{10}e_{\mathrm{sat}}=\;-7.90298\;(\frac{373.16}{T}-1)+5.02808\log_{10}\frac{373.16}{T}-1.3816\times 10^{-7}(10^{11.344(1-\frac{T}{373.16})}-1)+8.1328\times 10^{-3}(10^{-3.49149(\frac{373.16}{T}-1)}-1)+\;\log_{10}(1013.246)$$

Abbreviations: *e*_sat_, saturation water vapour pressure (hPa); log, logarithm in base 10; *T*, temperature in Kelvin.

#### Absolute humidity equation (H_A_)


$$H_{A}=\frac{m_{v}}{V}$$

$$e=\;H_{A}R_{v}T\;(\text{law of ideal gases})$$

$$H_{A}=\;\frac{e}{R_{v}T}$$

Abbreviations: *e*, water vapour pressure (Pa); *m_v_*, mass of water vapour (g); *H_A_*, absolute humidity (kg/m^3^); *R_v_*, 461.52 (J kg^−1^K^−1^) specific gas content of water; *T*, temperature in Kelvin; *V*, mass of dry air in a certain volume of air at a specific temperature (m^3^).

### Relative cooling effect

The efficacy of each device is represented in the relative cooling effect (by percentage). The perfect cooling system will show a 100% relative cooling effect. The relative cooling effect was calculated by dividing the difference between the temperature within the cooling device (internal temperature) and the external temperature by the maximum cooling effect. To estimate the maximum cooling effect, subtracting the wet bulb temperature from the external temperature was calculated. Therefore, wet bulb temperature, which is the lowest temperature that the evaporation of water can cool air, was determined by empirical expression functions [16,17].

$$\text{Wet}\;\text{bulb}\;\text{temperature}=T\;\times \text{arctan}[0.151977\times \;{\left(\text{rh}\%\;+\;8.313659\right)}^{1/2}]\;+\;\text{arctan}\left(T\;+\;\text{rh}\%\right)\;-\;\text{arctan}\left(\text{rh}\%\;-\;1.676331\right)\;+\;0.00391838\times {\left(\text{rh}\%\right)}^{3/2}\times \;\text{arctan}(0.023101\times \;\text{rh}\%)\;-\;4.68603$$

Maximum cooling effect (°C) = wet bulb temperature − external temperature

Relative cooling effect (%) = [(external temperature − internal temperature) ÷ maximum cooling effect]×100

Abbreviations: arctan, arctangent; rh, relative humidity; T, temperature in Celsius.

### Statistical analysis

All analyses were conducted using Stata Statistical Software version 16.0 (StataCorp LLC, College Station, TX, USA). Data of temperature and humidity was shown in mean and standard deviation (*SD*). The temperature difference was analyzed using a mixed-effects linear regression to compare external temperature and internal temperature. The constant ability to reduce temperature was analyzed using a mixed-effects logistic regression. Statistical significance was defined as *p*-value <.05.

## Results

### Inter-thermometer variability

Individual thermometers were ranged from −0.13 to +0.17 °C (*SD* = 0.07 °C). The deviance was small, which was interpreted as all thermometers being highly accurate and reliable.

### Temperature and humidity results

**Figure 2.**
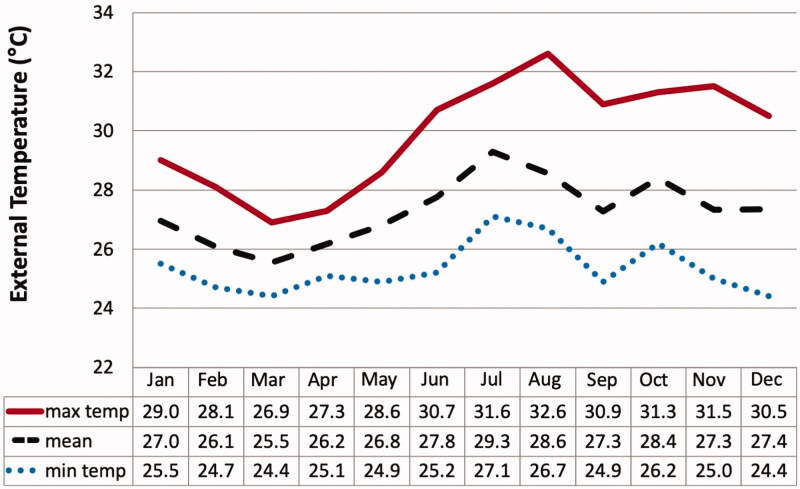
Mean external temperature in 12-month period.

There is a total of 52,020 records of raw temperature and humidity in this study. The mean external temperature was 27.3 ± 1.5 °C throughout the year (Figure 2). Mean humidity was 78.2 ± 7.1%RH. The highest external temperature and humidity were 32.6 °C in May and 92.1%RH in December. The lowest temperature and humidity were 26 °C in November and 48.2%RH in March.

**Figure 3.**
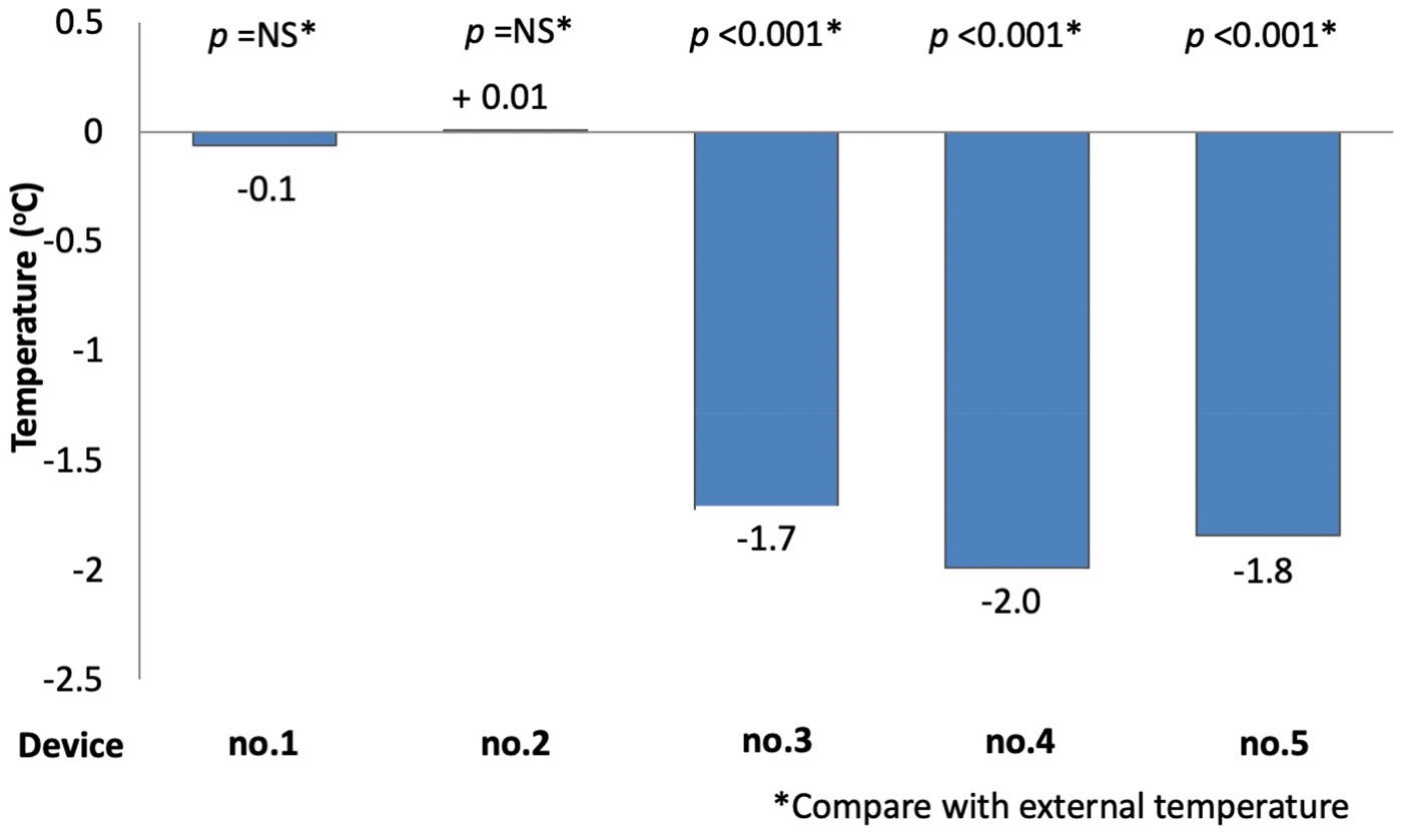
Temperature reduction achieved by each cooling device.

**Figure 4.**
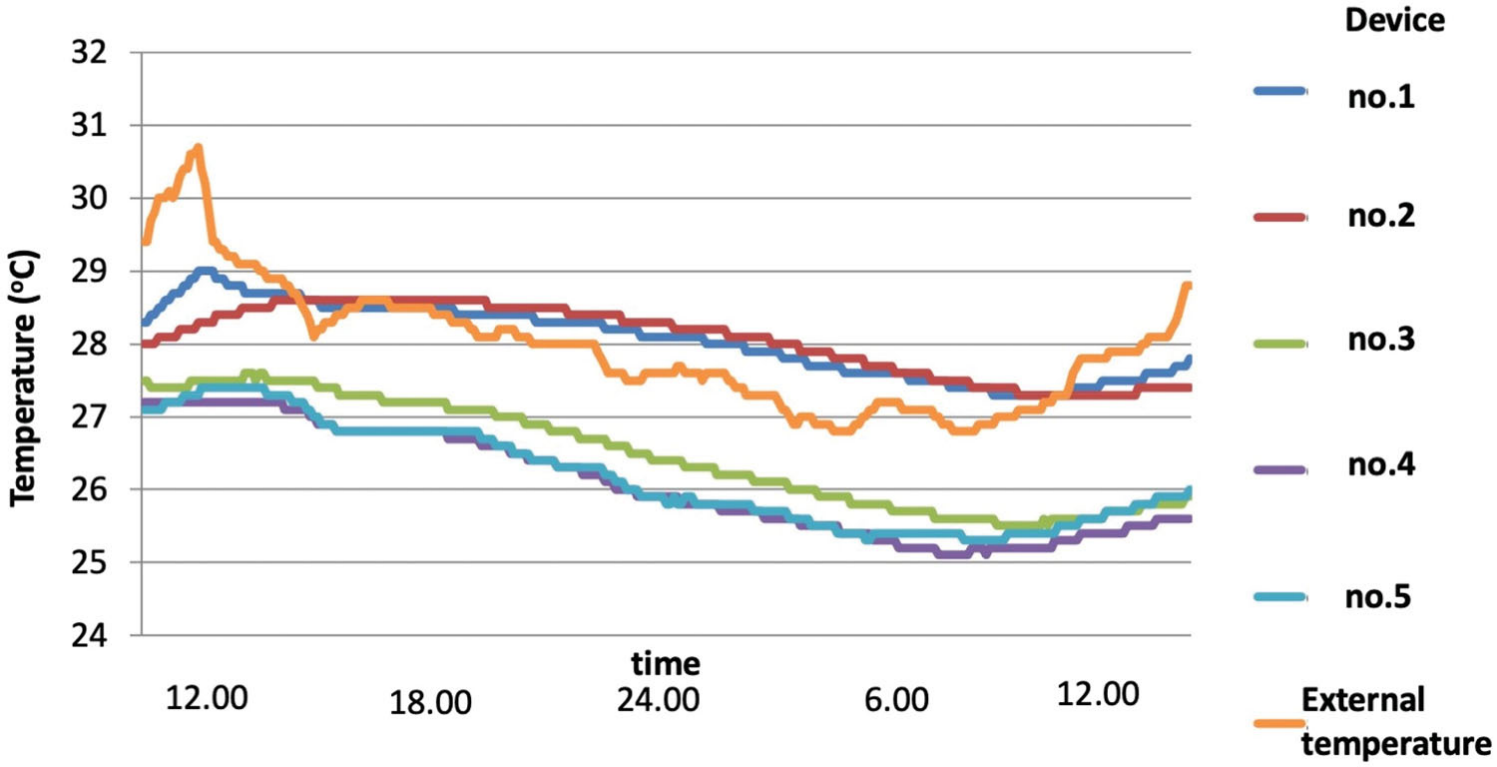
Temperatures in each cooling device and external temperature.

The mean differences between the external and internal temperatures were; device (1) −0.1 ± 0.6 °C (*p*=NS); (2) 0.0 ± 0.8 °C (*p*=NS); (3) −1.7 ± 0.9 °C (*p* < .0001); (4) −2.0 ± 0.9 °C (*p* < .0001); and (5) −1.8 ± 0.9 °C (*p* < .0001). Devices no. 3, 4, and 5 achieved significantly reduced temperature when compared with external temperature (Figures 3, 4). In contrast, devices no. 1 and 2 did not reduce the temperature but increased temperature compared with external temperature.

Saturated water vapour pressure were; device (1) 30.1 ± 2.3 Pa, (2) 31.1 ± 2.2 Pa, (3) 28.5 ± 1.8 Pa, (4) 28.1 ± 1.7 Pa, and (5) 27.7 ± 1.8 Pa, respectively (*p* < .001). Absolute humidity were; device (1) 21.7 ± 1.5 g/m^3^, (2) 22.4 ± 1.5 g/m^3^, (3) 20.6 ± 1.3 g/m^3^, (4) 20.4 ± 1.2 g/m^3^, and (5) 20.1 ± 1.3 g/m^3^, respectively (*p* < .001).

Maximal temperature reduction in each cooling device when compared with external temperature were: device (1) −2.4 °C, (2) −3.1 °C, (3) −5.4 °C, (4) −6.1 °C, and (5) −5.5 °C, respectively. The temperature reduction of device no. 4 ranges from −0.1 to −6.1 °C. Devices no. 1 and 2 could not maintain internal temperature lower than external temperature throughout the testing period. Only devices no. 3, 4, and 5 mostly achieved constant temperature reduction compared with external temperature (Figure 4).

**Figure 5.**
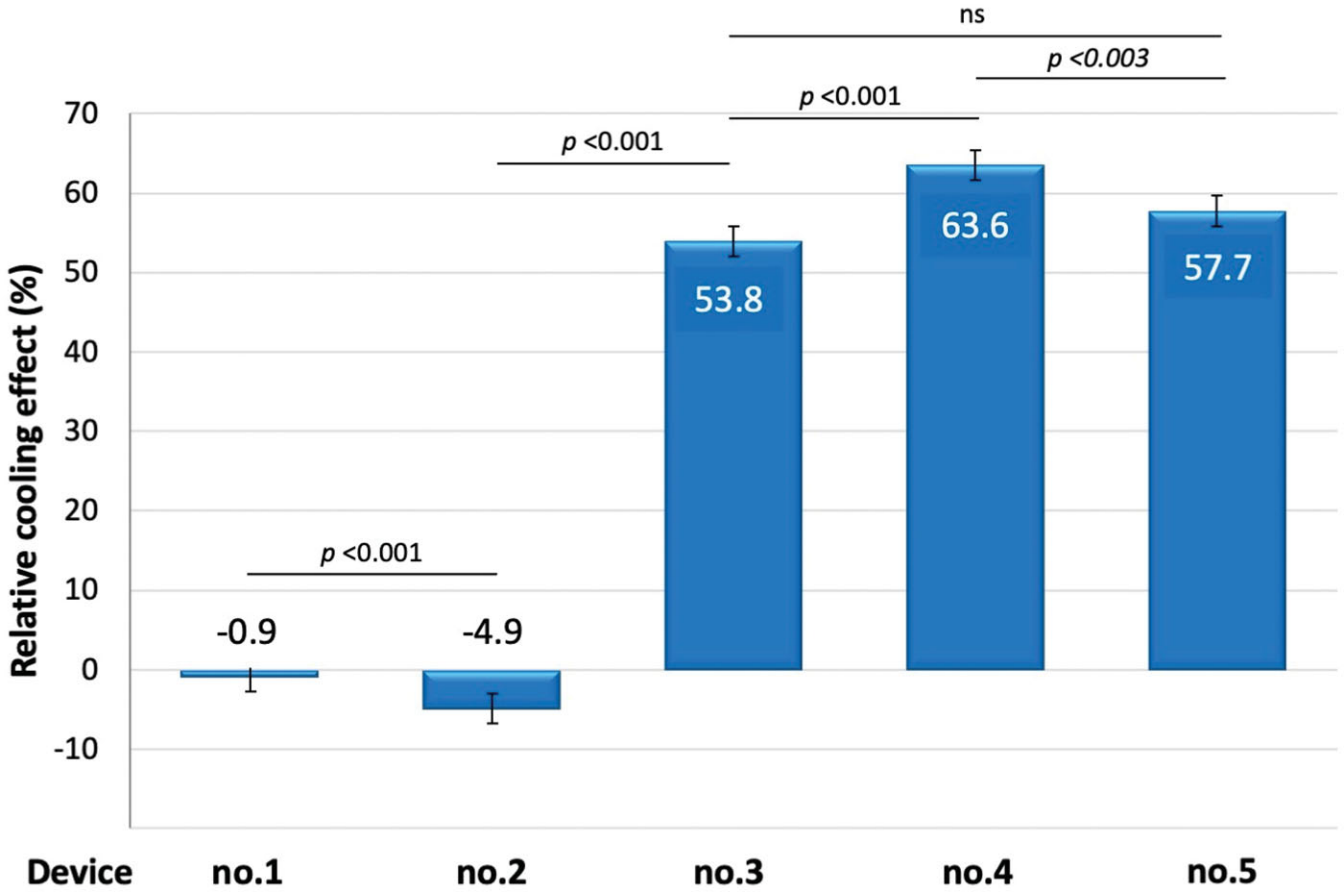
Relative cooling effect for each cooling device.

The comparison of the relative cooling effect was presented in Figure 5. Relative cooling effect of each device (*p*-value for the differences between each device and device no. 5) were; device (1) −0.9 ± 20.1% (*p* < .01); (2) −4.9 ± 25.3% (*p* < .001); (3) 53.8 ± 16.7% (*p*=NS); (4) 63.6 ± 14.3% (*p* < .003); and (5) 57.7 ± 12.2%. The two clay pots, gap filled with wet sand (device no. 4) exhibited significantly superior cooling efficacy when compared to device no. 5 with *p* < .003. Device no. 1 and 2 showed a significantly lower relative cooling effect than device no. 5.

To assess the constant ability of temperature reduction in each device, the percentage of achievement lowest temperature was recorded as shown in Table 2. Devices no. 3, 4, and 5 achieved constant temperature reduction compared with the external temperature at a temperature lower than 30 °C about 100% and mostly achieved constant temperature reduction at a temperature lower than 28 °C. In comparison with device no. 5, device no. 4 was superior in reducing the temperature (*p* < .001) and better achieved constant ability temperature at 25 °C of external temperature.

**Table 2.** Percentage of temperature achievement.

Temperature	<25 °C	<28 °C	<30 °C
Device no. 1	0.8%	71.4%	98.2%
Device no. 2	0.1%	70.9%	98.3%
Device no. 3	25.4%	99.5%	100%
Device no. 4	37.1%*	99.3%	100%
Device no. 5	30.3%	99.3%	100%

*In comparison with device no. 5, device no. 4 was superior to reducing the temperature (*p* < .001) and better achieved constant ability temperature at 25 °C of external temperature.

## Discussion

This study demonstrated that two traditional low-cost devices (two clay pots with wet sand or soil-filled in the gap) and a commercially manufactured cooling wallet used for insulin storage were efficacious in reducing insulin storage temperature in a hot-humid environment. The two clay pots with wet sand filled in the gap were the most efficacious device, with a temperature reduction efficacy of 2 °C and a relative cooling of 63.6%.

A Zeer-pot refrigerator or a pot-in-pot refrigerator is a traditional food cooler that applies the evaporative cooling technique to keep food fresh without electricity. It consists of two clay or terracotta pots, which the smaller inner pot holds the stuff. The gap between the pots is filled with sand and water, which slowly percolates to the outer surface of the pots and transfers heat from the inner pot, creating a cooling effect. The wet sand also functions as thermal insulation keeping the inner pot cool. A previous study in Saudi Arabia demonstrated that a Zeer consisting of an unglazed clay pot closed with a clay lid could reduce the temperature by ∼12 °C [15]. There was no change in insulin bioactivity and safety after storing insulin in a Zeer in the desert for up to six weeks. In contrast to the study in South Africa, the reduction of temperature from a porous unglazed clay pot is only 0.8 °C [18]. The differences in water vapour pressure and absolute humidity might underlie the contradictory results. A study in Saudi Arabia had more temperature reduction than in South Africa because the experiment was performed in a desert area which had low water vapour pressure and absolute humidity [15]. However, none of these previous studies assessed humidity [15,18] until Ogle et al. confirmed that the cooling effects were decreased in the high humidity environment [16]. Our study was conducted in the Southern area of Thailand, which had high humidity. Consequently, our clay pots had lower temperature reduction efficacy than the studies in dry regions [16,18].

In Thailand, a porous glazed earthen jar is the popular traditional water container to keep rainwater cool for household consumption. This storage jar was first developed in China and used as a container for long-distance maritime trade between the ninth and eighteenth centuries. These storage jars are usually covered by a lid to preserve grains and liquids. Local guidelines about disaster preparedness recommend keeping insulin storage in the earthen jar during the period of a power outage. This is the reason why we choose the earthen jar in this experiment. However, the porous glazed earthen jars covered with aluminium lids were not effective cooling devices because of high absolute humidity. Similar to the previous study, some devices covered with lids were less efficacious due to the limitation of evaporation [16]. In addition, the glazing prevents heat transfer and evaporation [16]. Two clay pots with wet sand or soil filled in the gap and FRIO^®^ commercially cooling wallet were more efficacious in evaporation. Clay pots with wet sand or soil are constructed using the pot-in-pot refrigerator concept. They have greater evaporation due to porosity without glazing and covering lids. Also, the sand and soil between pots can act as insulators and have higher thermal conductivity than water [19]. FRIO^®^ wallet is also an evaporative cooler. After immersion of the FRIO^®^ wallet in the water for 5–15 min, crystals in the wallet will expand into a gel, which remains cool for up to several days.

There are several options of cooling devices for use in a remote areas. A study in Sudan found that goatskin was one of the most efficacious devices to reduce temperature [16]. Despite the high efficacy, it is not practical to use goatskin as a cooling device in tropical countries because animal skin can cause unpleasant smells in hot-humid weather conditions. Therefore, the choice of insulin storage should be considered according to the availability of materials and certain circumstances. A clay pot is a simple and inexpensive cooler made with locally available materials and practically used in many countries, including Thailand. Regarding contamination, we advise putting the insulin vial in a clean plastic bag, placing it in dry condition, and not storing the insulin vial submerged in water. We compared traditional devices with FDA-approved commercially cooling wallets for insulin storage devices (FRIO^®^, device no. 5). Clay pots with sand or soil exhibited comparable cooling efficacy and sustainability with the FDA-approved cooling wallet. However, this commercial cooling wallet is more expensive and available only in some regions. Recently, the more affordable homemade wallet made of a hand-sewn cotton pouch and water beads demonstrated comparable cooling efficacy with the FRIO^®^ cooling wallet [20].

While clay pot is efficacious, the EADSG guidelines recommend avoiding keeping insulin in clay pots due to the high probability of contamination [1]. The injection site abscesses can be caused by injecting contaminated insulin stored in a water container [6]. When a refrigerator is not working, insulin should be stored according to the manufacturer’s instructions in a clean container at room temperature.

According to pharmaceutical manufacturers’ instructions, unopened insulin vials should be stored in the refrigerator within a range of 2–8 °C to guarantee their full effectiveness until the expiry date. When opened, insulin can be stored at a temperature of 25–30 °C but should then be used within 4 weeks (range 3–8 weeks, depending on the product). In one study on the thermostability of insulin, the potency of human insulins decreased by 14 and 18% when stored at 32 and 37 °C for 28 days, respectively [5]. However, a recent study in a refugee camp in Kenya showed that the structural and efficacy of various insulin (rapid-acting, NPH, and mixed insulins) could be preserved after the 28-day exposure to oscillating temperatures ranging between 25 and 37 °C [21]. These results showed that insulins could be stored safely even at warmer temperatures than previously recommended. However, further research is needed to explore insulin thermostability, particularly in settings of longer duration than 4 weeks and higher temperatures.

The strengths of this study include the measurements of temperature and humidity were performed with many traditional cooling devices and a commercial device under the simultaneously same conditions. These experiments permitted a standardized assessment of the relative cooling effect. The study was conducted in a real-life situation in a family home and used traditional low-cost devices which can be found easily in the local area of Thailand. Experiments were repeated multiple times throughout the year. However, the maximal external temperature did not reach the previously recorded hottest temperature in Thailand. By the way, the reported temperature in our study may not truly represent the external temperature because we recorded the indoor temperature with no direct sunlight. Our study also has several limitations. First, many cooling devices were not included in this study. Second, we did not investigate the influence of floor temperature, evaporative surface area, the shape of the devices, the volume of water, and the type of soil or sand effect on optimizing cooling. Third, we did not measure the quality of insulin after storage in a cooling device. Therefore, further studies on insulin stability are needed to determine when these devices are necessary.

In conclusion, traditionally low-cost devices, such as clay pots reduce storage temperatures to or close to room temperature in hot-humid climates. Although the refrigerator remains the standard of insulin storage, these evaporative coolers without electricity could be the alternative option for insulin storage in a limited-resource situation of tropical regions.

## Data Availability

The data that support the findings of this study are available from the corresponding author upon reasonable request.
